# Crusted lesions on the face of a child

**DOI:** 10.1111/pde.13648

**Published:** 2018-08-21

**Authors:** Yu‐Mei Li, Hui Xu, Hong Ma, Zhi‐qiang Chen

**Affiliations:** ^1^ Department of Dermatology The Affiliated Hospital of Jiangsu University Zhenjiang China; ^2^ Laboratory for Regeneration Medicine Jiangsu University Zhenjiang China

A 13‐year‐old girl presented with a 2‐month history of erythematous papules, blisters, erosions, and crusting on her face and ears. The patient had been previously diagnosed with erythema multiforme or contact dermatitis and treated with antihistamines, antiviral agents, and other medications, but without obvious effects. The patient stated that the lesions first appeared on her ears with no obvious predisposing trigger, and gradually spread to her cheeks after 2 months. On physical examination, the patient exhibited extensive erythema, papules, blisters, and some bullae on her face and ears. Some blisters had burst and left thick eschars, especially on her forehead and cheeks (Figure 1A‐B). There was a small amount of scattered erythema on her neck, trunk, and distal limbs, with no obvious blisters (Figure 1C‐D). There was no mucosal involvement, sore throat, cough, or expectoration. She had a history of joint pains in her feet but no known drug allergies nor remarkable familial genetic history.

**Figure 1:**
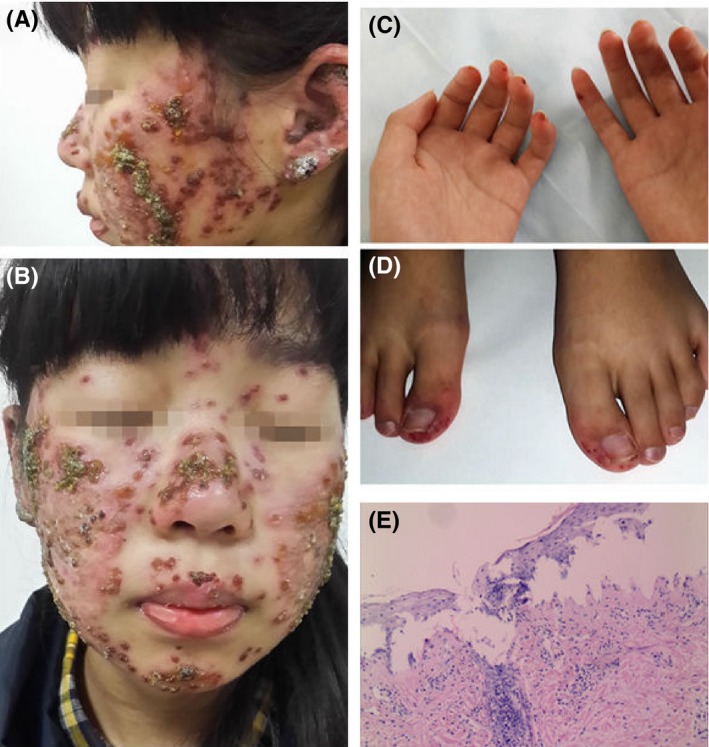


Upon examination, the blood test revealed decreased red blood cells (3.43 × 10^12^/L), peripheral white blood cells (2.7 × 10^9^/L), and platelet counts (90 × 10^9^/L), with elevated alanine transaminase (ALT) (123 U/L), aspartate transaminase (AST) (88 U/L), lactate dehydrogenase (LDH) (424 U/L), and adenosine deaminase (ADA) (35.6 U/L). The serologic test revealed positive antinuclear (1:1000 titer; particulate pattern) and anti‐dsDNA (123.4 IU/mL) antibodies, as well as anti‐Sm, RNP/Sm, SS‐A, ribonucleo‐P protein, and nucleosome antibodies. Serum C3 was low at 0.38 g/L (NR, 0.8‐1.7 g/L), as was C4 at 0.08 g/L (NR, 0.2‐0.6 g/L). A skin biopsy was taken (Figure 1E).

## WHAT IS THE DIAGNOSIS?

### Diagnosis: Systemic lupus erythematosus

 

## DISCUSSION

**Figure 2:**
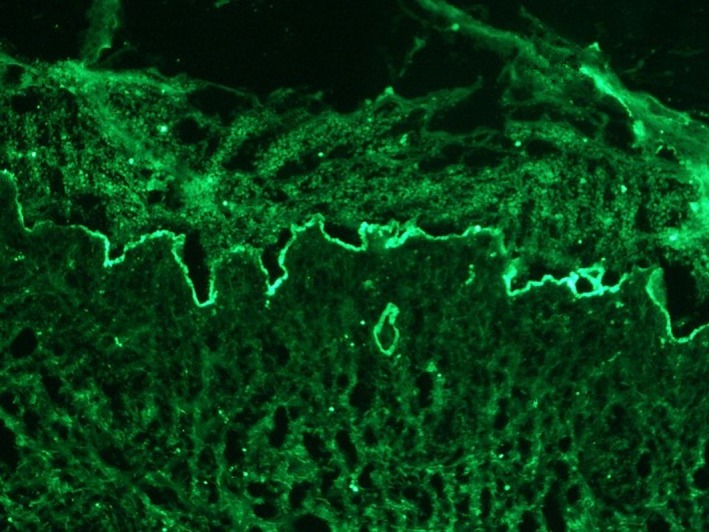


Based on the clinical manifestations and serologies, a diagnosis of systemic lupus erythematosus (SLE) on the face was suspected.^1^ Skin biopsy showed subepidermal blisters, liquefaction degeneration of basal cells, and visible neutrophil and lymphocyte infiltration around the upper dermis, hair follicles, and appendages (Figure 1E). Direct immunofluorescence (DIF) showed linear positive granular immunoglobulin G (IgG), IgM, and C3 deposition in the epithelial basement membrane zone (BMZ) (Figure 2). Systemic methylprednisolone at 0.75 mg/kg/qd and oral colchicine were administered. A topical therapy combining fusidic acid with erythromycin ointment was prescribed. The lesions gradually improved, and no new rash appeared.

Systemic lupus erythematosus is an autoantibody‐mediated system disease.^1^, ^2^ In some cases, skin lesions of SLE may exhibit vesicles, bullae, and erosions with crusts overlying erythematous edematous plaques. Bullous lesions in SLE, also called bullous SLE (BSLE), are mainly located on sun‐exposed areas, such as the head, face, trunk, and upper limbs, but they are more common on the trunk. Blister lesions can present as pemphigoid‐like eruptions with tense vesicles and bullae; alternatively, they are clustered like dermatitis herpetiformis; Nikolsky's sign is usually negative.^3^, ^4^ The diagnosis of BSLE requires the demonstration of autoantibodies against the BMZ protein, type VII collagen, or an immune electron microscopy examination revealing immunoglobulin deposition under the lamina densa and reticular lamina.^5^, ^6^

Our patient showed certain remarkable features. First, the disease was consistent with a diagnosis of SLE. Second, the skin lesions were characterized by acquired noncicatricial bullae; skin biopsy histopathology showed a subepidermal blister with neutrophil and lymphocyte infiltration around the upper dermis and basement membrane zone. Direct immunofluorescence showed positivity for a linear, granular, or mixed linear and granular pattern of IgG deposition on the skin lesions and surrounding areas or accompanied by IgM and/or IgA deposition. However, in the absence of obvious bullous lesions, autoantibodies against the BMZ protein, type VII collagen were not tested, and immune electron microscopy examination was not carried out, a diagnosis of BSLE cannot be attained.

In patients with LE, erythema multiforme (EM)‐like lesions with positive tests for speckled antinuclear antibody have been reported under the name Rowell syndrome (RS).^7^, ^8^ In our patient, EM‐like lesions did not coexist continuously with LE, and thus, a diagnosis of Rowell syndrome was deemed unlikely.

Our patient presented a rare manifestation of SLE, with vesicular lesions that were mainly concentrated on the face, accompanied by Raynaud's phenomenon. We reviewed the related literature and found no related previous reports. The present case indicates that this condition represented with a rare manifestation of SLE and its precise pathogenesis remains to be investigated.

## FUNDING INFORMATION

This article was supported by Project in Zhenjiang (No. SS2015024), and the National Natural Science Foundation of China (No. 81573053).
